# The effect of CBT and its modifications for relapse prevention in major depressive disorder: a systematic review and meta-analysis

**DOI:** 10.1186/s12888-018-1610-5

**Published:** 2018-02-23

**Authors:** Zuojie Zhang, Lingli Zhang, Guorong Zhang, Jianing Jin, Zhenyang Zheng

**Affiliations:** 1 Department of Pharmacy, Evidence-based Pharmacy Center, West China second hospital, Sichuan University, Chengdu, China; 2 Key Laboratory of Birth Defects and Related Diseases of Women and Children (Sichuan University), Ministry of Education, Chengdu, China; 3 West China School of Pharmacy, Sichuan University, Chengdu, China; 4 Department of Neurology, Fujian Medical University attached Union Hospital, Fuzhou, China; 5 West China Second University Hospital, Sichuan University, No.20,Third Section, Renmin Nan Lu, Chengdu, Sichuan 610041 People’s Republic of China; 6 Chengdu City, China

**Keywords:** major depressive disorder, remission, relapse, cognitive behavioural therapy

## Abstract

**Background:**

The risk of relapse in major depressive disorder (MDD) is associated with high worldwide disease burden. Cognitive behavioral therapy (CBT) and its modifications might be effective in relapse prevention. The aim of this review was to evaluate the efficacy of these treatments for reducing relapse of MDD.

**Methods:**

The retrieval was performed in the databases of MEDLINE via Pubmed, EMBASE and PsycINFO via OVID, The Cochrane Library and four Chinese databases. Clinical trials registry platforms and references of relevant articles were retrieved as well. Hazard ratio (HR) and corresponding 95% confidence interval (CI) were used to pool evidences.

**Results:**

A total of 16 eligible trials involving 1945 participants were included. In the first 12 months, CBT was more efficacious than control in reducing the risk of developing a new episode of depression for MDD patients in remission (HR:0.50, 95%CI:0.35–0.72, I^2^ = 11%). Mindfulness-based cognitive therapy (MBCT) was more efficacious than control only among patients with 3 or more previous depressive episodes (HR:0.46, 95%CI:0.31–0.70, I^2^ = 38%). Besides, compared with maintenance antidepressant medication (m-ADM), MBCT was a more effective intervention (HR:0.76, 95%CI:0.58–0.98, I^2^ = 0%). These positive effects might be only maintained at two and nearly 6 years follow up for CBT.

**Conclusion:**

The use of CBT for MDD patients in remission might reduce risk of relapse. Besides, the effect of MBCT was moderated by number of prior episodes and MBCT might only be effective for MDD patients with 3 or more previous episodes. Further exploration for the influence of previous psychological intervention is required.

**Electronic supplementary material:**

The online version of this article (10.1186/s12888-018-1610-5) contains supplementary material, which is available to authorized users.

## Background

Major depressive disorder (MDD) is one of the most common and prevalent mental disorders, which is characterized by low vigor, low mood, low self-confidence, and aversion to activity without a specific reason [1, 2]. It is one of the leading causes of worldwide disability and is associated with approximate 16% lifetime prevalence rate [3, 4]. Besides, it is related with continuous high risk of recurrence which represents an increased disease burden [5, 6]. Following each new episode, the condition of depression becomes worse and risk of next relapse increases [7, 8]. Researchers found that risk of relapse after experiencing one episode of major depression was 50%, after two was 80% and after three might be up to 90% [9, 10].These relapses are associated with considerable high cost to individual, family, and society [6, 11]. Therefore, in view of the nature of depression-related impairments and future implication of recurrent depression, attempting to prevent the relapse of depression is an important clinical therapeutic goal for long-term management of MDD.

The most commonly used treatment for reducing depression relapse rate after successful pharmacotherapy might be sequential pharmacotherapy [12]. But long-term use is associated with enormous side effects such as drug interaction [13, 14]. In addition, even if the discontinuation reaction is gradual [15], patients who reluctant to continue pharmacotherapy are possible to relapse when discontinue the medicine [16]. In clinical practice, sequential pharmacotherapy is associated with high risk of noncompliance [17, 18] and psychotherapy is chosen with intense patient preference [19]. A research found that preference was a powerful influence for the effect of intervention [20]. Compared to unfavorable intervention, favorable intervention brings more positive results to patient [21].In addition, different from pharmacotherapy which might be invalid in the phase of discontinuation, psychotherapy brings with potential long-term benefit [22]. The lasting effect may be attributed to the fact that these patients could either improve the cause of recurrent risk or understand many useful techniques to hold back the risk of relapse [23]. As a result, looking for alternative suitable psychotherapy is a public health priority.

Cognitive behavioral therapy (CBT) is one of the most frequently used psychosocial treatment for mental disorders, which targets at changing tactic of patients to cope problems in cognitions (such as belief and thought) and behaviors [24, 25]. Different kinds of modifications of CBT were found to be effective as well, such as mindfulnessbased cognitive therapy (MBCT) [26–28]. MBCT uses conventional methods of CBT which combined with mindfulness meditation. It is a class-based skills training program and developed for increasing the ability of patients to prevent the recurrence of depression in the long term [29]. MBCT could not only help patients to be more aware of negative thoughts in the period of potential relapse but also allow them to get rid of rumination after depression [30]. Through MBCT, therapists could empower participants to process their experiences via mindfulness and meditation skills, and thus participants could improve their undesirable feelings [31].

However, many investigators found that MBCT might not reduce the risk of developing a new relapse of depression in patients with 2 previous episodes [28, 32]. Investigators offered two hypotheses. The first one was that depressive thoughts were derived from repeated connections between the depressed condition and negative thinking modes. The enhancement of these connections with higher numbers of episodes was thought to result in increasing the risk of relapse after each episode. The increased risk of relapse for patients with 3 or more previous episodes was assumed to be attributable to autonomous relapse processes involving reactivation of depressogenic thinking patterns by dysphoria. For patients with higher numbers of episodes, less environmental stress is needed to motivate relapse [33]). The preventative effect of MBCT was assumed to attribute to disrupt these processes at times of potential relapse by decreasing the extent of depressive thought reactivated by negative feelings [28, 34]. The second was that there might be different categories of depression. One category might be associated with reaction to life events, namely among patients with fewer numbers of previous episodes. Another category of depression might be associated with heightened rumination, namely among patients with a higher number of previous episodes [28].

Previous researches paid attention to the role of psychotherapy on the prevention of relapse. Jacob Piet and Esben Hougaard reported their researches in 2011 which investigated the effect of MBCT for preventing relapse or recurrence among patients with MDD in remission, but some comparisons included few evidences [35]. Currently, the incremental number of clinical trials make it a necessary to conduct an update metaanalysis. Katherine Clarke and colleagues identified the efficacy of all nonpharmacological interventions for preventing further episode, but they did not evaluate the influence of number of previous episodes and they defined control arms as any intervention, which might bring clinical heterogeneous [28, 32, 36]. The study by van der Velden et al. only investigated mechanism of MBCT in the treatment of recurrent MDD [37]. In light of the above evidences and the unclear comprehensive effectiveness of CBT and its modifications for reducing relapse rate in subjects with MDD in remission (a specific period of MDD), we conducted this review and meta-analysis to extend prior studies. The purpose of this review was to describe relevant research in this topic, and then to evaluate the short and long term efficacy of CBT and its modifications. In addition, in order to evaluate the influence of number of previous episodes, we separately analyzed the efficacy of trials which only included patients with 3 or more than 3 previous episodes.

## Methods

Overall, our review was performed in line with the PRISMA statement [38, 39].

Inclusion and exclusion criteria.

The selection criteria was formulated as follows:

1. Patient: Aged ≥18 years old with MDD in full or partial remission based on a strict diagnostic definition, such as Diagnostic and Statistical Manual of Mental Disorders (DSM) or International Classification of Diseases 10th revision (ICD- 10). Full remission is defined as a relative brief period during which the individual is asymptomatic. Asymptomatic is not defined as a complete absence of symptoms but instead is defined as no more than minimal symptoms. Asymptomatic is operationalized as a score of < 8 on the 17-item Hamilton Depression Rating Scale (HDRS). Partial remission is defined as persons whose depression do not meet the full remission criterion but who have clinical meaningful reductions in baseline, which refers to a HDRS-17 score<12 [40, 41].
2. Intervention: All CBT and its modifications (such as CBT, cognitive therapy(CT), behavioral therapy(BT), Cognitive Behavioral Analysis System of Psychotherapy (CBASP), and MBCT) will be selected regardless of their different modalities(face-to-face, Internet, or other), formats (group or individual), number of sessions, duration of each session, and frequency.
3. Comparison: Any comparator intervention, including control (treatment-as-usual (TAU), (psychological or pill) placebo (PLA), wait-list (WL), and psychoeducation) and maintenance antidepressant medication (m-ADM).
4. Study design: Randomized controlled trials (RCT).

Furthermore, the eligible articles were limited to report in English or Chinese.

Discontinuation researches were excluded.

### Search strategy and study selection

The retrieval of eligible studies was performed from 1976 to September 1, 2016 in the following databases: MEDLINE via Pubmed, EMBASE and PsycINFO via OVID, The Cochrane Library, Chinese Biomedical Literature Database (CBM), China Knowledge Resource Integrated Database (CNKI), VIP Database and Wanfang Database. References of relevant articles were retrieved manually. In addition, unpublished trials were retrieved with the help of International Clinical Trials Registry Platform (ICTRP) (http://www.who.int/ictrp/search/en/) and clinical trial registries platform (http://clinicaltrials.gov/). The search used all relevant terms of ‘depression’, ‘relapse’, and ‘cognitive behavioural therapy’, with a limit to ‘randomized controlled trial’. Additional file 1 showed a detailed systematic search strategy.

After removing duplicate articles, two investigators reviewed the remaining articles independently according to the selection criteria and decided whether the full-text reports should be reviewed. And then, after reviewing the eligible full-text reports, studies which met the selection standard were included in our review.

Disagreements were discussed and resolved by joining a third investigator.

### Data extraction and quality assessment

Two investigators independently extracted the following data through a pre-designed Excel table: basic information (surname of the first investigator, year of publication, baseline relapse status of depression, the criteria used to measure relapse, inclusion and exclusion criteria, pharmacological interventions used before enrolment), characteristics of participations (sample size at randomization and dropout, age, sex, proportion of ADM used at baseline, mean age of first onset), interventions and comparisons (explicit definition, form of intervention, number of sessions, duration of each session, frequency, length of follow-up). Two investigators independently assessed risk of bias of individual studies according to the methods in Cochrane Handbook [42].

Any missing data were requested from some authors through e-mail to replenish our analysis. A third investigator checked the consistency of extractions and coordinated any discrepant data.

### Statistics analysis

In our meta-analysis, we defined outcome measure as hazard ratio (HR) and corresponding 95% confidence interval (CI) of developing a new episode of depression. If study reported HR with 95%CI, these data would be recorded preferentially. If not and only Kaplan-Meier curve or observed event data could be reached, HR with 95%CI would be calculated through survival plots or observed event data according to the method provided by Tierney et al. [43]. For outcome at the specific time (such as the results at 12 months or 24 months in the follow up), we preferred to get the designated HR from Kaplan-Meier curves as we could.

Statistical heterogeneity of pooled HRs was examined by Q test and I^2^ statistics. *P* < 0.1 or I^2^ > 50% demonstrated that there was substantial heterogeneity. If that, we would identify potential factors which generated heterogeneity (clinic, methodology, and statistics). The random-effect model was chosen to pool HRs. Separate meta-analyses were conducted among: 1) different follow-up durations (12 months, 24 months, and longer than 24 months); 2) different kinds of comparison (control arm and maintenance antidepressant medication (m-ADM)); 3) different numbers of prior episodes (subjects with < or ≥3 previous episodes). Pre-designed subgroup analyses of different kinds of CBT were carried out. Because CBT and CT were essentially the same treatment but with some minor differences, we did not distinguish them and treated CT as CBT. In our meta-analysis, control arm was defined as treatment-as-usual condition. Studies with other kinds of comparator were only included in narrative analysis and excluded from statistics meta-analysis.

Funnel plots were chosen to test publication bias if any of the separate outcome included 10 or more trials as recommended by Cochrane Handbook [42], because the study number less than 10 was thought to be lack of statistical power to receive a reliable result. All the above statistical analyses were performed with the help of Review Manager 5.

## Results

### Studies included

Our search initially reached 53 articles which potentially met the inclusion and exclusion criteria. Finally, a total of 20 eligible articles (16 trials with 1945 participants, sample sizes ranged from 40 to 424) were included in our narrative review [26–28, 32, 44–59], of which 18 articles were included in our meta-analysis. Two were failed to be included because of either inappropriate control arm or lacking sufficient relapse data [56, 57]. One trial was conducted by Stangier et al., which was a multicenter prospective randomized observer-blinded study. Patients (*n* = 180) with three or more previous MDD were assigned to maintenance CBT or manualized psychoeducation. Analysis found that maintenance CBT was only significantly more efficacious than manualized psychoeducation in subjects with five or more previous episodes. Because the control arm was manualized psychoeducation, which did not meet our criterion of statistics analysis for meta-analysis. We excluded this trial from meta-analysis. Another trial was by Teismann et al., in which participants (*N* = 60) were randomly assigned to either CBT or wait-list control condition. Although the aim of this study was to investigate whether CBT group treatment was effective in reducing residual depression by targeting depressive rumination, authors reported outcome of relapse as secondary outcome. Authors found that CBT was effective for depressive rumination. For the relapse prevention, they only reported that one person (3.2%) suffered from a relapse in the first 6 months and eight persons (25.8%) suffered a relapse within the first year. We failed to calculate HR from these data, so we excluded this study from our meta-analysis as well.

**Fig. 1:**
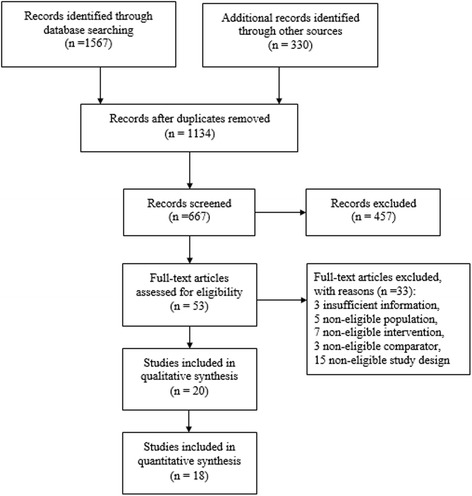
Flow chart of meta-analysis

PRISMA flow chart was depicted in Fig. 1.

The characteristics of included 16 trials were depicted in Table 1. The mean age was 46.8 (range: 24–75). For the outcome measure, apart from one trial by Teismann et al., all these trials used relapse as primary outcome measure. For all the modifications of CBT, our retrieval found that only MBCT has been investigated for reducing relapse rate in subjects with MDD in remission.

**Table 1:** Characteristics of included trials

Study ID		Pre randomization /eligibility				Patients						Interventions			
Author	Year	Condition	Previous episodes	Criteria used to measure relapse	Previous pharmacology interventions	N	Age^a^	Male (N/%)	Previous episodes^a^	% on ADM at baseline	Mean age of first onset^a^	Groups (n/dropouts)	Form of intervention	N(Number of sessions, frequency, Mean duration + boost sessions)	Length of Follow-Up(months)
Bockting	2005	recurrent MDD, in remission for 10 weeks - 2 years	2 + recent episodes in the previous 5 years	SCID-I	NR	187	44.7/9.5	46/27	3.5/3.8	51	28.4/12.5	CBT:97/16	group	8,weekly,2 h	24
												Control(TAU):90/6			
Bondolfi	2010	recurrent MDD, in remission for 3 + months	3 + episodes (2 in the past 5 years and at least one in the past 2 years)	SCID	a history antidepressants treatment but off medication for 3 + months before enrolment.	60	47.5(24–66)	17/28	4(3–14)	30	24.5(8–55)	MBCT:31/4	group	8,weekly,2 h + 4 sessions each 3 months	14
												Control(TAU):29/1			
Fava	1994	primary MDD, in full remission	NR	RDC-defined MDE	successfully response to antidepressant drugs according to a standardized protocol	40	43.7/2.3	16/40	NR	100	NR	CBT:20/0	individual	10,every other week,40 min	24
												Control(PLA):20/0			
Fava	1998	MDD, in full remission for 10 week +	3 or 3 + episodes	RDC-defined MDE	successfully response to antidepressant drugs according to a standardized protocol.	40	46.1/3.7	13/32.5	3.6/0.8	100	NR	CBT:20/0	individual	10,every other week,30 min	24
												Control(PLA):20/0			
Godfrin	2010	MDD, in remission for 8 + weeks	3 or 3 + episodes	DSM-IV diagnosis of MDE	No special	106	45.7/10.5	20/19	NR	76.4	29.8/10.2	MBCT:52/18	group	8,weekly, 2.75 h + homework exercises (meditation practices) for 45 + min/d	14
												Control(TAU):54/12			
Hollandare	2013	MDD, in partial remission	1 + episodes in the previous 5 years	SCID	No special	84	45.3/12.8	13/15	5.96/9.1	50	NR	CBT:42/10	individual	16 internet modules + general e-mails for 10 weeks	24
												Control(PLA)::42/7			
Kuyken	2008	recurrent MDD, in full or partial remission	3 or 3 + episodes	SCID	on a continuing dose of m-ADM for 6 + months	123	49.2/11.2	29/24	6.4/3.0	100	26.2/12.1	MBCT-TS:61/9	group	8,weekly,2 h + 4 sessions	15
												m-ADM:62/10			
kuyken	2015	recurrent MDD, in full or partial remission	3 or 3 + episodes	SCID	on a continuing dose of m-ADM for 6 + months	424	49.5/12.5	99/23	46% 6 +	100	24.9/12.4	MBCT-TS:212/23	group	8,weekly, 2.25 h + 4 refresher sessions/3 months	24
												m-ADM:212/18			
Ma and Teasdale	2004	MDD, in remission for 12 + weeks	2 or 2 + episodes in the previous 5 years	modeled on the SCID	a history antidepressants treatment but off medication for 3 + months before enrolment.	75	44.5/9.0	18/24	3/2.0	100	30.6/12.4	MBCT:37/6	group	8,weekly, 2.75 h + homework exercises	13
												Control(TAU):38/1			
Paykel	1999	MDD, in remission for 2–18 months	residual symptoms	DSM-III-R criteria for MDD for at least 1 month AND Minimum symptom score	all on a history antidepressants treatment	158	43.4/10.5	80/51	37% 3 +	100	NR	CBT:80/19	individual	16 sessions over 20 weeks + 2 sessions 6 and 14 weeks later	15.8
												Control(PLA):78/12			
Segal	2010	MDD, in remission for 7+ months	3 + episodes	16 + on the HRSD-17 assessed twice then criteria for MDD measured with SCID	all on a history antidepressants treatment	54	44.1/11	31/37	4.7/2.3	55	31/11.6	MBCT:26/5	group	8,weekly,2 h + option 1 h/month mindfulness mediation class	18
												m-ADM:28/7			
Stangier	2013	MDD, in full remission	3 or 3 + episodes	DSM-IV diagnosis of MDE (LIFE)	NR	180	48.6/11.6	50/27.8	7.4/8.3	74.8	30.9/12.4	Maintenance CBT: 90/15	individual	16,NR,50 min	12
												Manualized psychoeducation: 90/31	individual	16,NR,20 min	
Teasdale	2000	MDD, in remission(0-24 months)	3 + episodes (2 in the past 5 years and at least one in the past 2 years)	SCID	a history antidepressants treatment but off medication for 3 + months before enrolment.	145	43.3/10.3	35/24	3.3/3.0	100	26.8/10.2	MBCT:76/13	group	8,weekly,2 h + 4 more sessions over trial duration	12
												Control(TAU):69/0			
Teismann	2014	MDD, in partial remission	NR	SCID	No special	60	47.1/11.8	17/28	22.45% 5 +	40	NR	CBT:31/2	group	11,weekly,90 min	12
												WL:29/5			
Wilkinson	2009	MDD, in remission for 2 + months	NR	MADRS score of 10 + at 6/12 months	Received antidepressant medication	45	74.0/7.3	17/38	3.0/1.9	100	NR	CBT:22/4	group	8,NR,90 min	12
												Control(PLA):23/5			
Williams	2014	MDD, in remission for 8+ weeks	3 or 3 + episodes	SCID	NR	164	43.4/12	76/28	77% 5 +	44	21.3/10.7	MBCT:108/9	group	individual pre class interview + 8,weekly,2 h + follow-up classes taking place 6 weeks and 6 months in the follow-up	12
												Control(TAU):56/3			

Note:

^a^ Mean/SD, Median(range)

*CBT* cognitive behavioral therapy, *CPE* cognitive psychological education, *MBCT* mindfulness-based cognitive therapy, *MBCT-TS* MBCT with support to taper or discontinue antidepressant treatment, *TAU* treatment as usual, *m-ADM* Maintenance antidepressant medication, *WL* wait-list, *PLA* (psychological or pill) placebo

Criteria used to measure relapse:

*DSM* Diagnostic Statistical Manual, *LIFE* Longitudinal Interval Follow-up Evaluation, *MDD* MDD Disorder, *MDE* MDD Episode, *RDC* Research Diagnostic Criteria, *SCID* Structured Clinical Interview for DSM Disorders, *MADRS* Montgomery–Asberg Depression Rating Scale, *HRSD* Hampton Roads Sanitation District

## CBT

There were many differences in CBT manuals of included CBT trials, including mediums (face-to-face, Internet, or other), formats (group or individual), number of sessions, duration of each session, and frequency. In addition, the inclusion criteria of eligible patients, such as previous interventions of these patients, in these trials were different as well.

Two trials by Fava et al. [46, 49] implemented the intervention of CBT as described by Beck et al. [60], while others respectively used their own manuals which were modified from that by Beck et al. [60]. All the authors delivered CBT in face-to-face modality other than Holländare et al. [52], who delivered it in the modules of internet communication and encrypted e-mails which largely decreased time of therapist. Apart from three trials which delivered CBT in the format of group [26, 57, 58], others delivered in the format of individual. The explanation to group format was that it was more cost-effective and patients included were current free of psychopathology [26]. Sessions ranged from 8 to 16 and durations ranged from 30 to 90 min.

## MBCT

In general, in contrast to CBT, the therapeutic manual, mediums, number of sessions, and duration of each sessions of MBCT in included MBCT trials were mainly consistent, namely eight weekly 2-h group training sessions in line with the manual published by Segal, Williams, & Teasdale [31], while the adjunction sessions had several unconformities among trials as displayed in Table 1. In order to eliminate the influence of previous other psychological interventions, all MBCT trials excluded patients with concurrent psychotherapy or more than one psychiatric consultation per month, four of which unequivocally excluded patients undergoing more than four sessions of CBT ever or positive response to CBT [28, 32, 45, 59].

### Meta-analytic results

#### 12 months

##### CBT and its modifications VS control

**Fig. 2:**
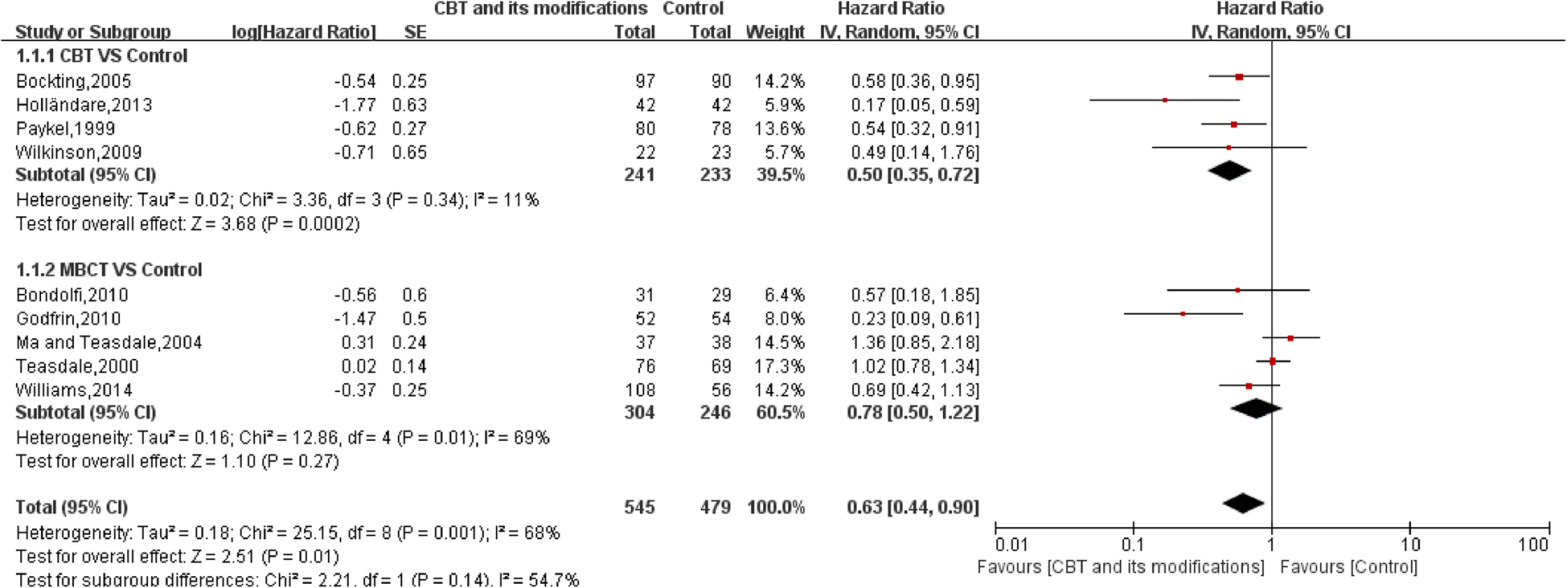
Relapses in 12 months in CBT and its modifications vs Control

This separate meta-analysis included 9 trials. Compared with control, risk of depression relapse was reduced by 37% for CBT and its modifications (HR: 0.63, 95%CI:0.44–0.90) (Fig. 2).

#### CBT VS control

This subgroup included 4 trials. Compared with control, risk of depression relapse was reduced by 50% for CBT (HR:0.50, 95%CI:0.35–0.72, I^2^ = 11%).

One trial by Bockting et al. [26]. randomized 187 recurrent depressive patients in remission for 10 weeks to 2 years to either brief CBT plus control (*N* = 97) or control(*N* = 90), with continuing use of medication in both groups. Patients who recently received CBT or other psychotherapies were excluded. One was a medium trial by Hollandare et al. [52], which compared the efficacy of Internet-based CBT (16 CBT-based modules via a secure internet communication platform plus encrypted e-mail communication with a personal therapist, *N* = 42) to control group (only the e-mail communication, *N* = 42) for reducing the risk of relapse in partially remitted depressive patients. Medication was allowed to receive. In the trial conducted by Paykel et al. [54], 158 major depressive subjects who partially remitted from receiving antidepressant for at least previous 8 weeks were randomized to either 16 sessions of CBT for 20 weeks or control. Antidepressants were continued and maintained in all subjects. The last trial was conducted by Wilkinson et al. [58], they randomized 45 patients with MDDs in remission for at least 2 months to brief group CBT (CBT-G)(*N* = 22) or control(*N* = 23).

#### MBCT VS control

There were 5 trials among this subgroup. Compared with control, overall HR did not show a significant preventive effect for MBCT(HR:0.78, 95%CI:0.50–1.22, I^2^ = 69%) with high heterogeneity (Fig. 2).

Of these trials, three, respectively conducted by Bondolfi et al. [45], Ma and Teasdale [32], and Teasdale et al. [28], were largely identical in inclusion/exclusion criteria and treatment framework. For the administration of medication, in the section of enrolment, subjects were required to receive antidepressants previously but discontinue them for at least 3 months before inclusion, and in the section of experiment, some of the antidepressants were reinstated.

For the study by Godfrin et al. [51], it randomized 106 MDD patients in remission for at least 8 weeks to MBCT or control. As to medication such as antidepressants, subjects were permitted to receive.

Williams and colleagues [59] conducted a three arm trial (MBCT, cognitive psychological education and control). As to the use of medication in experimental stage, subjects were encouraged to continue their administrations at enrolment.

##### CBT and its modifications VS m-ADM

**MBCT VS m-ADM**

**Fig. 3:**
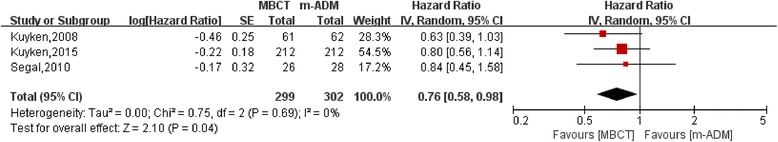
Relapses in 12 months in MBCT vs m-ADM

Pooled HR from three trials showed that MBCT was associated with a lower risk of relapse rate than m-ADM at 1 year follow-up (HR:0.76, 95% CI:0.58–0.98, I^2^ = 0%) (Fig. 3). Two trials were reported by Kuyken and colleagues in 2008 (pilot trial) and 2015, respectively. They evaluated whether MBCT plus taper or discontinue antidepressant treatment (MBCT-TS) outperformed m-ADM in preventing depressive relapse or recurrence. Before the enrolment, all the subjects received a maintain antidepressant dose according to the British National Formulary (BNF) and NICE guidance. The last trial was by Segal et al. [55], in which MBCT (*n* = 26) and m-ADM (*n* = 28) were randomized to remitted depressive patients with at least 3 past episodes.

#### 24 months

##### CBT and its modifications VS control

**CBT VS Control**

**Fig. 4:**
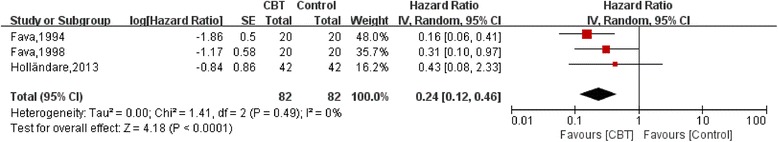
Relapses in 24 months in CBT vs Control

There were 3 trials among this subgroup. Compared with control, risk of depression relapse was reduced by 76% for CBT (HR:0.24, 95%CI:0.12–0.46, I^2^ = 0%)(Fig. 4).

One trial by Hollandare et al. [52] was described above. The remaining two trials (number of subjects both were 40) were all conducted by Fava and colleagues. The first one (1994) aimed at evaluating the efficacy of CBT for treating residual symptoms of primary major depressive and the other one (1998) was conducted in patients with recurrent depression (at least 3 episodes of depression). All the subjects were in full remission after successfully receiving standardized antidepressant. And then they were randomly assigned to either CBT or control (both with discontinuing ADM).

##### CBT and its modifications VS m-ADM

**MBCT VS m-ADM**

For this comparison, only one trial conducted for 24 months [27]. Compared to m-ADM, MBCT did not show a significant relapse prevention effect (HR:0.89, 95%CI:0.67–1.17).

#### Longer than 24 Month

Three trials reported efficacy of CBT for preventing relapse at more than 24 months follow-up.

Bockting et al. [44] followed up their trial for 5.5 years and reported the results, which showed that there was a significant relapse preventive effect of CBT for MDD patients(HR:0.71, 95%CI:0.52–0.97) and the effect reinforced with the number of previous episodes. More narrowly, compared to control, patients with 4 or more previous episodes showed a more significant effect for CBT(HR:0.46, 95%CI:0.28–0.75), and patients with less than 4 previous episodes showed a non-significant effect (HR:0.86, 95%CI:0.51–1.45).

The other two trials by Fava et al. [48, 50] were followed-up for 6 years, which found that compared to control, CBT had a significant relapse prevention effect at 6-years follow-up (HR:0.36, 95%CI:0.18–0.72). Of note, one of these two trials reported the 4-year follow-up result [47], which showed that CBT was associated with a lower relapse rate than control (HR:0.45, 95%CI:0.16–0.97) as well.

#### Number of prior episodes

**CBT VS Control**

Only one trial by Fava et al. (1998) [49] reported subjects with 3 or more previous MDD episodes. They found that compared to control, CBT had a preventive effect (HR:0.31, 95%CI:0.10–0.97).

**MBCT VS Control**

**Fig. 5:**
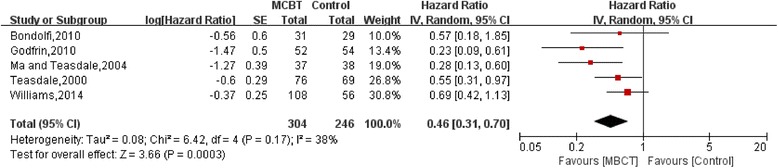
Relapses in MBCT vs Control for participants with three or more previous episodes of major depression

Apart from two trials by Ma and Teasdale [32] and Teasdale et al. [28], all trials of MBCT only included MDD patients with 3 or more previous episodes. These two also included subjects with 2 previous episodes and separately evaluated relapse rates in the subgroup of patients with < or ≥3 previous episodes. Pooling data of patients with 3 or more previous episodes together, we found that compared to control arm, MBCT reduced the risk of developing a new depressive episode by 54% (HR:0.46, 95%CI:0.31–0.70, I^2^ = 38%) (Fig. 5).

##### Quality assessment

Risk bias of the included 20 studies were displayed in Table 2. The majority of studies reported methodology of random sequence generation (*N* = 15) and allocation concealment (*N* = 13), while others provided insufficient information, leading to “unclear risk”. Owning to the characteristic of psychotherapy, all the studies had a high risk of bias in the blinding of participants and personnel. 15 studies (75%) were judged to be prone to a low risk of bias in blinding of outcome assessment. 60% studies had a low risk of incomplete outcome data, while others had unclear or high risk due to either insufficient information or high scale of dropout. ITT analyses were available for 11 studies, while others only reported results of completer analyses. 6 studies (30%) were judged to be high risk of selective reporting.

**Table 2:** Risk of bias in included studies

Study ID	Random sequence generation	Allocation concealment	Blinding of participants and personnel	Blinding of outcome assessment	Incomplete outcome data	Selective reporting	Other sources of bias
Bockting, 2005 [26]	L	L	H	L	U	H	L
Bockting,2009 [44]	L	L	H	L	U	H	L
Bondolfi,2010 [45]	L	L	H	L	L	L	L
Fava,1994 [46]	U	U	H	L	L	L	L
Fava,1996 [47]	U	U	H	L	L	L	L
Fava,1998 a [48]	U	U	H	L	L	L	L
Fava,1998 b [49]	U	U	H	L	H	L	L
Fava,2004 [50]	U	U	H	L	L	L	L
Godfrin,2010 [51]	L	L	H	H	U	L	L
Hollandare,2013 [52]	L	U	H	U	L	L	L
Kuyken,2008 [53]	L	L	H	L	L	L	L
Kuyken,2015 [27]	L	L	H	L	L	L	L
Ma and Teasdale,2004 [32]	L	L	H	L	L	H	L
Paykel,1999 [54]	L	L	H	L	U	H	L
Segal,2010 [55]	L	L	H	L	U	H	L
Stangier,2013 [56]	L	U	H	H	H	H	L
Teasdale,2000 [28]	L	L	H	L	L	L	L
Teismann,2014 [57]	L	L	H	H	L	L	L
Wilkinson,2009 [58]	L	L	H	L	U	L	L
Williams,2014 [59]	L	L	H	H	L	L	L

Note: *H* high risk, *L* low risk, *U* unclear

##### Publication bias

As the explanation showed in Cochrane Handbook, visual examination analysis of funnel plots have limited power to detect bias if the number of studies is small [42]. Because any one of the separate meta-analyses did not cover 10 or more than 10 trials, we did not identify publication bias through visual inspection of funnel plots.

## Discussions

For short-term follow-up (12 months), our meta-analyses demonstrated that CBT was more efficacious in reducing the risk of developing a new relapse of depression than control, while compared to control, MBCT only showed a significant effect in patients with 3 or more previous depressive episodes. Besides, compared with m-ADM, MBCT was an effective intervention for relapse prevention. For long-term follow-up (24 or more than months), the preventive effect of CBT were maintained for 2 to 6 years in recurrent prevention. For MBCT, the only one trial found that MBCT and m-ADM were not significantly different from each other after 2 years in terms of relapse/recurrence.

For heterogeneity among comparisons, we thought it was associated with substantial differences in either the regimens of administration or the inclusion criterion regarding the use of psychotherapy or pharmacotherapy in MDD patients before enrolment.

Overall, our findings largely agreed with previous studies. Compared with the reviews by Beshai et al. and Bockting et al. [61, 62], our findings was from qualification of “best evidence” by using quantitative meta-analysis approach, which could illustrate the reliability and size of preventive effect of intervention on reducing relapse rate. Meta-analysis could also illustrate whether significant effect found in some studies are systematic or random. Our meta-analysis focused on evaluating the effect of CBT and its modifications for reducing relapse rate in subjects with MDD in remission (a specific period of MDD). This clinical question were more specific than the above two reviews. Therefore, the findings was more targeted. In addition, we used a different outcome measure of HR to summarize results. This measure has the advantage of applying more available information of the trial, including the number of patients who fail to complete the trial and time of patients occurring event in the duration of follow-up [63].

So far, two meta-analyses have been performed to evaluate the relapse prevention effect of psychotherapy in patients with MDD in remission [35, 36]. Compared with these two meta-analyses, we also found CBT to be effective in the prevention of a new depressive episode. The article conducted by Piet et al. [35] found that MBCT significantly reduced the risk of relapse compared to control and did not significantly reduce the risk of relapse compared to m-ADM. We reached different results. We found that compared to control, MBCT only significantly reduced the risk of relapse in patients with 3 or more previous episodes, not in all patients. It is to say that the effect was moderated by number of prior episodes. Compared to m-ADM, MBCT might have a preventive effect, but our effect size was at the edge(HR: 0.76, 95%CI:0.58–0.98). This inconsistency might because we included two more studies than this meta-analysis and used a different outcome measure. However, the evidences we included were insufficient to ascertain and relevant trials needed repetition to confirm or overturn our findings. Compared to another article by Clarke et al. [36] which did not evaluate the effect of MBCT at 2 years follow-up, we found that MBCT might not show a significant preventive effect at 2 years follow-up owning to our update retrieval. Although our analyses were somewhat similar to theirs which aimed to respectively display the effect of psychological interventions at 1 and 2 years follow up, the outcome measure we used could reach to these two particular time points which were thought to be more precise. The time points of outcome of the meta-analysis by Clarke et al. might be nearly 1 or 2 years and were vague. On the other hand, in their analyses, they mixed all control arms into meta-analysis without separate analyses, but we thought this brought clinical heterogeneity into results because these control arms were different from each other in clinic. Therefore, we evaluated them in separate analyses. In addition to these above differences, compared to these two meta-analyses, we also investigated the longer-term (more than 24 months) effect of CBT and MBCT, which haven’t be investigated in other previous meta-analyses.

There are some other strengths we want to display in our meta-analysis. To make sure the comprising of all relevant trials regardless of whether the results have been published and reduce the possibility of publication bias, we performed a comprehensive retrieval, especially including clinical trial registry platforms. To reduce random error which might generate from literature selection, data extraction, or quality assessment, two investigators took part in the preliminary stage and then a third investigator joined to check and coordinate the disagreements.

However, limitations were also required to be considered when interpreting our results. The main limitation was that the number of trials and sample sizes for some comparisons, especially MBCT versus m-ADM, were too small to address firm conclusion. Besides, our conclusion could not be popularized to patients in other status of depression because the inclusion criteria of our participants was MDD in full or partial remission. We excluded other patients to ensure the homogeneity as needed by meta-analysis. Finally, no formal protocol was set up at the start of this meta-analysis, although this research was conducted with specific pre-designed purpose and rigorous methods.

Some significance aspects were needed to be focused on in future studies. Firstly, we only displayed some information about previous interventions, including pharmacotherapy and psychotherapy, but failed to evaluate the influence of these interventions on result. Further studies are suggested to pay more attention to the influence of previous interventions, especially sequential psychotherapy interventions. Trials which reported efficacy result at long-term follow-up were still insufficient, and future researches are suggested to provide more data about this. Since preferences of patient and patient-specific clinical variations (such as preferences for particular psychotherapy in previous treatment and severity of every episode of depression) might moderate the effect of psychological interventions [20, 64], future researchers should notice that more flexible and available interventions are important and focus on how to choose better intervention for a particular individual. If these factors are considered, the potential effect of psychological strategies might enlarge. There is also need for trials with head-to-head comparisons of these different psychotherapies.

## Conclusion

There were evidences that for MDD patients in remission, CBT was an effective intervention for relapse prevention at either short or long term follow-up. Compared to control, MBCT demonstrated benefit in relapse prevention but only among subjects with 3 or more previous episodes, which meant that the effect of MBCT might be moderated by number of prior episodes. Further studies are suggested to pay more attention to evaluate the influence of previous interventions, especially sequential psychological interventions. There are also need for trials with psychological head-to-head comparisons.

## Additional file

Additional file 1: Results from the systematic search strategy. (DOC 36 kb)

## Abbreviations

ADM: Antidepressant medication
BNF: British National Formulary
BT: Behavioral therapy
CBASP: Cognitive Behavioral Analysis System of Psychotherapy
CBT: Cognitive behavioral therapy
CI: Confidence interval
CT: Cognitive therapy
HDRS: Hamilton Depression Rating Scale
HR: Hazard ratio
m-ADM: Maintenance antidepressant medication
MBCT: Mindfulness-based cognitive therapy
MBCT-TS: Mindfulness-based cognitive therapy plus taper or discontinue antidepressant treatment
MDD: Major depressive disorder
PLA: (Psychological or pill) placebo
RCT: Randomized controlled trials
