# Supplementary material for: The effect of CBT and its modifications for relapse prevention in major depressive disorder: a systematic review and meta-analysis
Source: BMC Psychiatry. 2018 Feb 23;18:50. doi: 10.1186/s12888-018-1610-5 (PMC6389220; doi:10.1186/s12888-018-1610-5)
Supplement: Supplementary file 1 — Results from the systematic search strategy. (DOC 36 kb) [file 12888_2018_1610_MOESM1_ESM.doc]

**Additional file 1**

Results from the Systematic Search Strategy*

| **Databases:** | **Citations** |
| --- | --- |
| MEDLINE | 431 |
| EMBASE | 442 |
| PsycINFO | 509 |
| The Cochrane Library | 41 |
| CBM | 37 |
| CNKI | 48 |
| VIP Database | 34 |
| Wanfang Database | 25 |
| Total (databases) | 1567 |
| Trial registers: International Clinical Trials Registry Platform (ICTRP) : <http://www.who.int/ictrp/search/en/>) and clinical trial registries (http://clinicaltrials.gov/) | 330 |
| Total | 1897 |

*Explicit search strategy : (title / abstract = depress* OR ‘depressive disorder’[MeSH Terms] OR ‘depression’ [MeSH Terms] ) AND (title / abstract = (recurrence* or relapse*) OR recurrence[MeSH Terms]) OR ‘Secondary Prevention’[Mesh]) AND (title / abstract = (psychotherap* or cognitive therap* or ‘behavioral therap*’ or ‘cognitive behavioural analysis system of psychotherap*’ or ‘mindfulness based cognitive therap*’)) AND ((randomized controlled trial[Publication Type]) or title / abstract = (random*))
